# Intravital skin ulcer in a late 18th century Spanish child mummy: evidence for septicemic death?

**DOI:** 10.1007/s12024-025-00959-z

**Published:** 2025-02-01

**Authors:** A. G. Nerlich, R. D. Loynes, A. Jardiel Badia, A. Begerock, D. Delgado, M. Gonzalez, Raffaella Bianucci

**Affiliations:** 1 Institute of Legal Medicine, Department of Forensic Histology, Paleopathology and Mummy Research, Ludwig-Maximilians-University, Nussbaumstr. 26, D-80336 Munich, Germany; 2 KNH Centre for Biomedical Egyptology, University of Manchester, Manchester, UK; 3 Museo de las Momias de Quinto, Zaragoza, Spain; 4 Institute for the Scientific Study of the Mummies (IECIM), Madrid, Spain; 5 Laboratory Anthropology, Archaeology, Biology (LAAB), Paris-Saclay University, 2 Avenue de la Source de la Bièvre, Montigny-Le-Bretonneux, 78180 France

**Keywords:** Histopathology, Infection, Sepsis, Church mummies, Spain

## Abstract

During excavation campaigns carried out in north-eastern Spain (Aragonese), a well -preserved late 18th century infant mummy was exhumed from the topsoil of El Piquete church (Quinto, Zaragoza). At morphological macroscopical observation, a penetrating lesion lateral to the right knee was identified. The lesion was covered by linen bindings with a circumscribed brownish discoloration. Investigations were carried out with a special focus on the lower tight lesion in order to ascertain whether it had occurred intra-vitam, perimortem or post-mortem. A CT scan was performed to establish the age at death of the infant and to identify possible pathological disorders of the skeleton and internal organs. Histology was performed on the ovoid, deep penetrating lesion in order to determine whether it was vital or was due to taphonomic alterations. The body belonged to a male, 12–16 months old infant. It did not show any pathological disorder apart from the presence of an ovoid deep penetrating skin lesion lateral to the right knee. Histology showed that, apart from the typical postmortem alterations, several small haemosiderin deposits, such as in siderophages, were present, thus indicating, not only the vitality of the ulceration, but also its age of more than several days. We conclude that the infant survived the traumatic lesion for a few days and he most likely died of systemic infection related syndrome (SIRS). This rare case adds to the paleopathological literature on children’s possible cause of death.

## Introduction

The interdisciplinary investigation of human remains offers a unique insight into life, disease and eventually death in past populations. The analysis of mummified remains even extends the potential information to numerous diseases that usually do not leave traces in the skeleton. While there have been numerous adult human mummies investigated from both intentional and non-intentional sources, only very limited information is available on infant mummies. This is mostly due to the fact that the preservation of infantile human remains is frequently limited due to their smaller size, the significantly higher fragility of the biomaterial, and less care during material recovery [1, 2]. Whilst soft tissue as well as bone of infants may perish very rapidly and completely under the usual conditions of human soil burials, bodies with artificial or spontaneous mummification [3] and protected storage conditions, such as in crypt burials, may result in preservation. These rare findings provide a unique insight into the infant´s life [4–8].

The present paper describes such a case of a very well preserved late 18th century infant mummy which was found in a larger burial setting within a Northeastern Spanish (Aragonese) church thereby permitting the preservation also of soft tissues and partly internal organs. The presence of an intravital ulceration of the skin strongly suggests severe local infection providing evidence for a potential final systemic inflammatory reaction syndrome (SIRS) thereby causing the death of the infant.

## Materials and methods

### The church and the crypt

During the restoration works in 2011 in the central nave of the church of the Assumption of Our Lady- known as “El Piquete”- in the village of Quinto (about 42 kms southeast of Zaragoza, Aragon, Spain), the human remains of 70 individuals were uncovered [9]. Originally built as a church fortress on the ruins of an old castle located on a hill named “The Crown”, the construction works commenced in the early 15th century and were completed in middle 18th century. Its current state is the result of different stages of remodelling, in which elements of the Gothic, Mudejar and Renaissance styles joined.

During two archaeological excavation campaigns (2011 and 2016–2017) a total of 158 burials were discovered within the church building, of which only 33 individuals presented different degrees of natural mummification (8 adults and 25 children) [9]. Currently, fifteen mummified bodies are located in the Museo de las Momias de Quinto. Based on archaeological and documentary sources, the bodies were dated between the mid-late 18th century (the first burial is dated 1760 CE) and mid-19th century (when a Royal decree had abolished burials within churches). All social classes are represented - ranging from wealthy peasants to high rank clerics. All mummified individuals showed preservation of various though dessicated and shrunken internal organs. Amongst these bodies, one infant mummy (PQ27) was of particular interest [9], since it presented with a significant skin defect on the right leg. The overlying fabric showed a brownish staining in the size of the defect supporting the notion of an intravital lesion. This case was further investigated.

### The infant mummy

The naturally mummified body of the child (PQ27) had been exhumed from the uppermost layer of the burials of El Piquete church in 2011 which suggests a burial period of roughly between 1760 and 1790 CE [9]. The body had been found in the residues of a wooden coffin of 70 × 30 × 20 cm size made of pine wood (*Pinus pinea L.*) in a typical truncated conical shape as usually used in the 18th century. The coffin was undecorated and there were no inscriptions which might have indicated the deceased´s name. Despite the availability of an extensive parish registry of the time period, the data did accordingly not allow the identification of the infant nor its family.

### Macroscopic and radiological evaluation

**Fig. 1:**
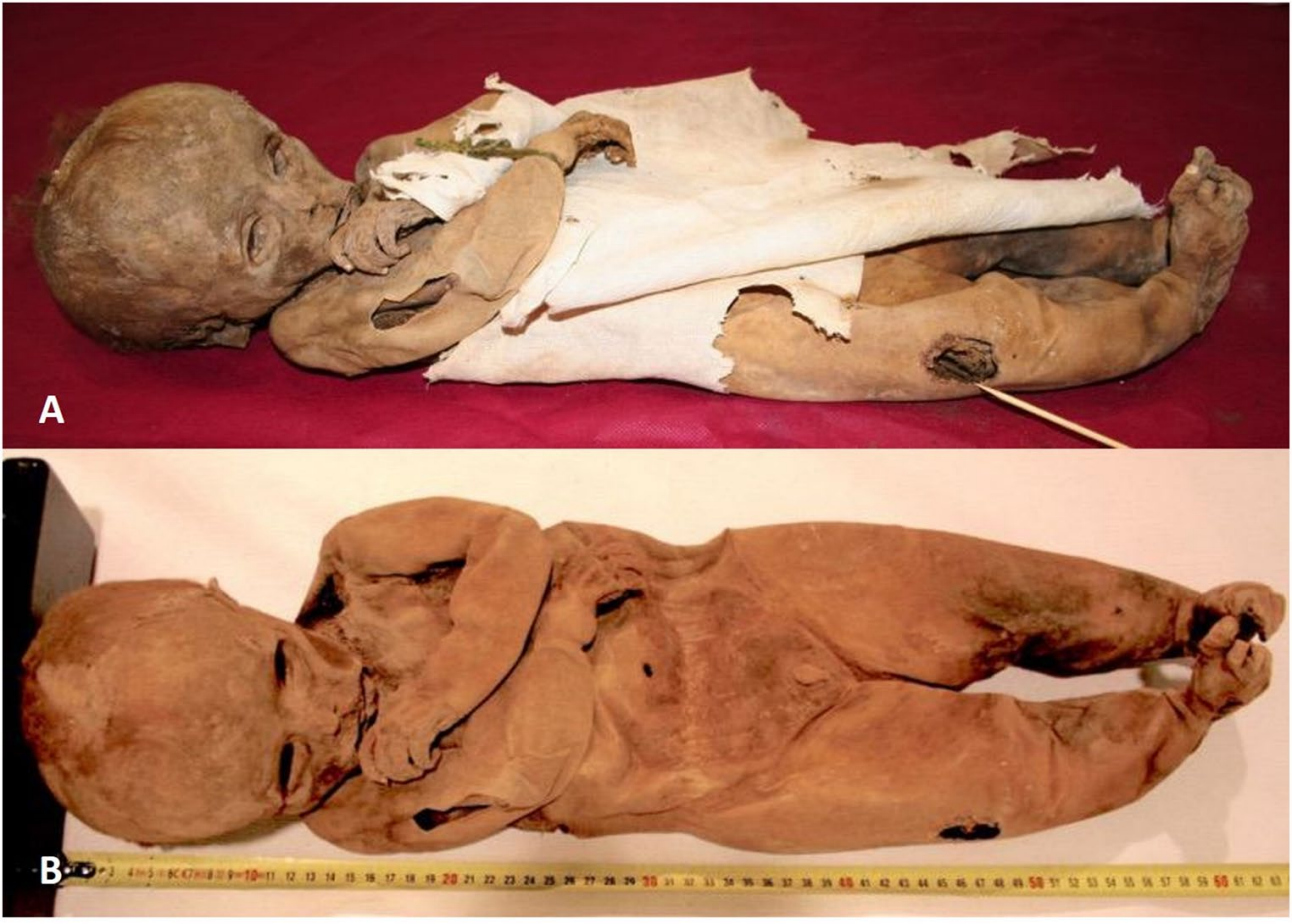
Macroscopic views of the child mummy immediately after removal from its coffin (**A**) and following removal of the fabric (**B**). Note the discoloration zone of the right leg

The infant´s body was covered by a long sheet of fabric with a smaller shirt below (see Fig. 1A). Firstly, the complete body size was determined. Following the macroscopic inspection the mummy was subjected to a whole-body CT-scan (Siemens Healthineers, Erlangen, Germany, slice thickness 1 mm, 120 kV/ 200 mA) in standard algorithm. The resulting data were evaluated on a power station with the opportunity for multiplanar reconstruction and 3D-rendering [10], in this case on an Apple iMac Retina 5K – 27”, 2020 using the operating system macOS 14.5 and Dicom Reader software Osirix MD v.14.0.1.

### Histological analysis

In order to obtain potential data for tissue rearrangement – and thereby about the vitality of the defect – a small 0.5 × 0.8 mm sample was obtained from the defect margin. This was rehydrated and embedded in an oriented position so that the ulceration surface was identified using a routine protocol as previously described [11]. The resulting paraffin block was used for histochemical stainings including haematoxylin & eosin (HE), Elastica-van Gieson connective tissue stain, periodic-acid Schiff´s reagent stain (PAS) and finally Prussian blue stain for the potential identification of haemosiderin deposits. All stainings followed established protocols [11].

## Results

### Macroscopic and radiological investigation

**Fig. 2:**
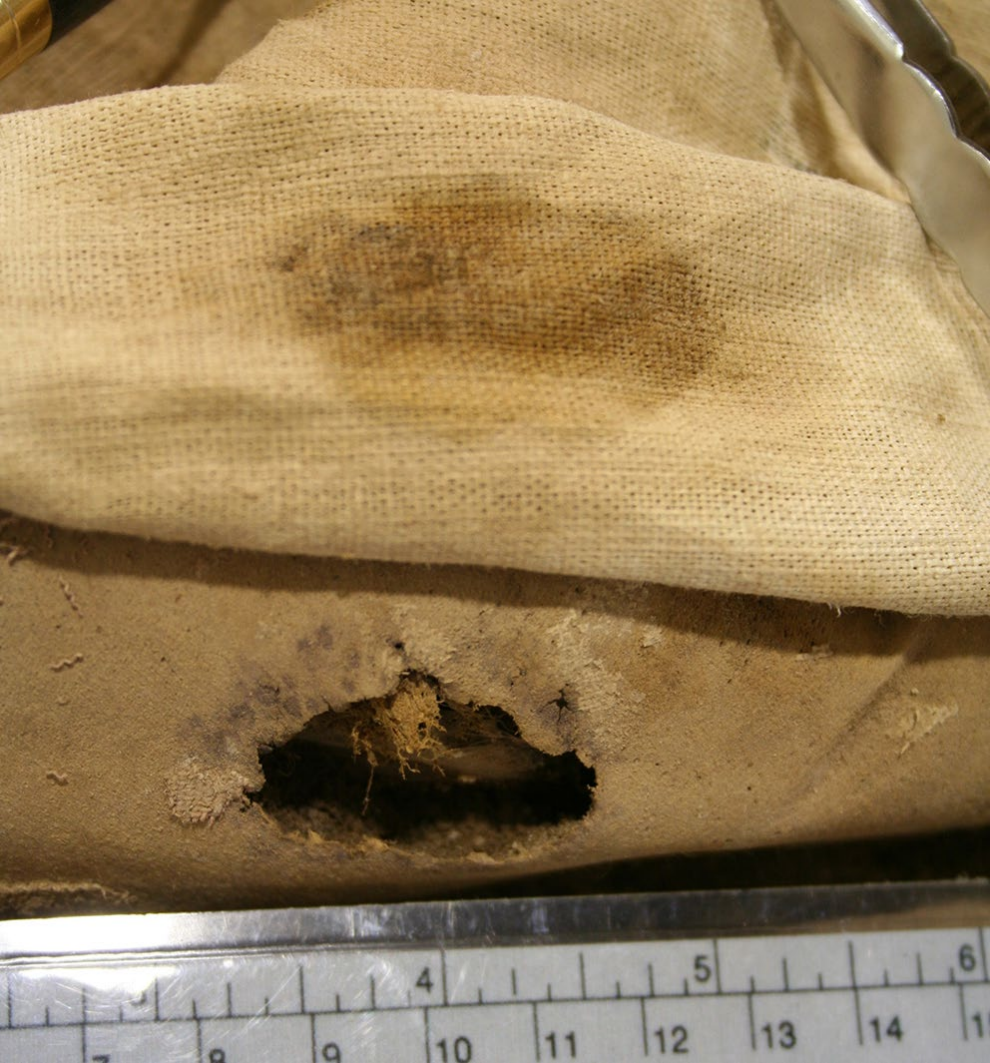
Close-up foto of the ulceration zone with the overlying fabric being tilted upwards. Note the round defect margins and the exact discoloration of the overlying fabric restricted to the defect zone

The child presented in supine position with its arms flexed in front of the ventral body wall (Fig. 1). External genitalia proved a male individual by the presence of penis and scrotum. The body had an overall length of ca. 61 cm, measurements of the extremities, the trunk and circumferences of head and chest showed proportionate values. The body was partly covered by a well-furnished fabric of cotton or linen. Most remarkably, over the knee region of the right leg a 2.2 × 3.6 cm large area of the fabric showed a brownish discoloration (see Fig. 1A). On further careful inspection and after raising the fabric, below this area a same sized ulceration of the skin could be detected with rounded fairly regular margins and a deeply penetrating defect zone of up to 14 mm depth (Figs. 1B, 2).

This defect has to be distinguished from a small longitudinally arranged skin laceration at the right upper arm of ca. 4 × 1 cm size which occurred during the further storage of the mummy obviously due to a rodent attack. At this site, there is no evidence for exudation or any other tissue reaction as seen at the afore described defect.

*.*

**Fig. 3:**
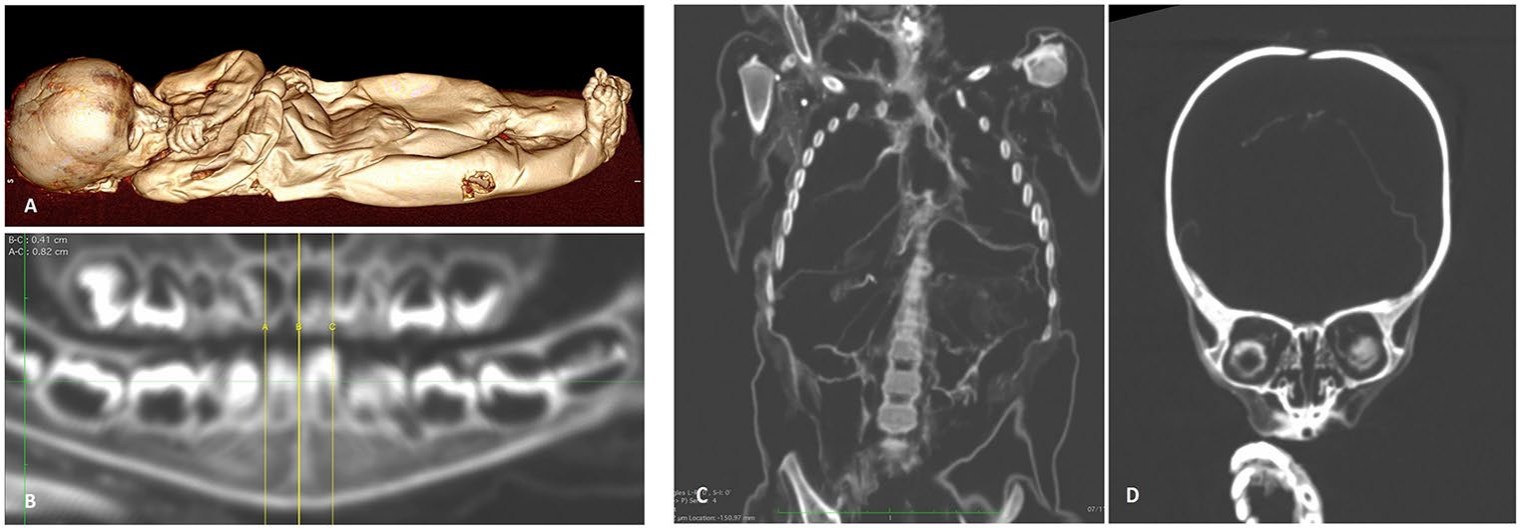
CT results of the infant mummy: (**A**) complete surface reconstruction highlighting the ulceration of the leg. (**B**) The dentition shows an individual age between 12 and 16 months. (**C**) Coronal plane through chest and abdomen with some residues of internal organs, but without pathological findings. (**D**) Coronal plane of the cranium showing residual tissue in the skull cavity and the remains of both eye balls

On CT scans the macroscopic findings are confirmed (Fig. 3A). Furthermore, the dentition could be evaluated showing an age at death of between 12 and 16 months (Fig. 3B) [12] which is in agreement to the overall body length. Further detailed evaluation reveals residues of internal organs, particularly in both thoracic cavities with the remains of both lungs shrunken and displaced to the dorsal chest wall (Fig. 3C). The abdominal cavity contains residues of otherwise not further identifyable organs due to postmortem degradation. At the cranial level, the meninges, some cerebral remnants and the desiccated eye globes could be appreciated (Fig. 3D). In summary, despite some post-mortem autolysis, the well preserved infant mummy does not provide any further pathological findings as to the internal organs preserved.

### Histological tissue analysis of the ulceration margin

**Fig. 4:**
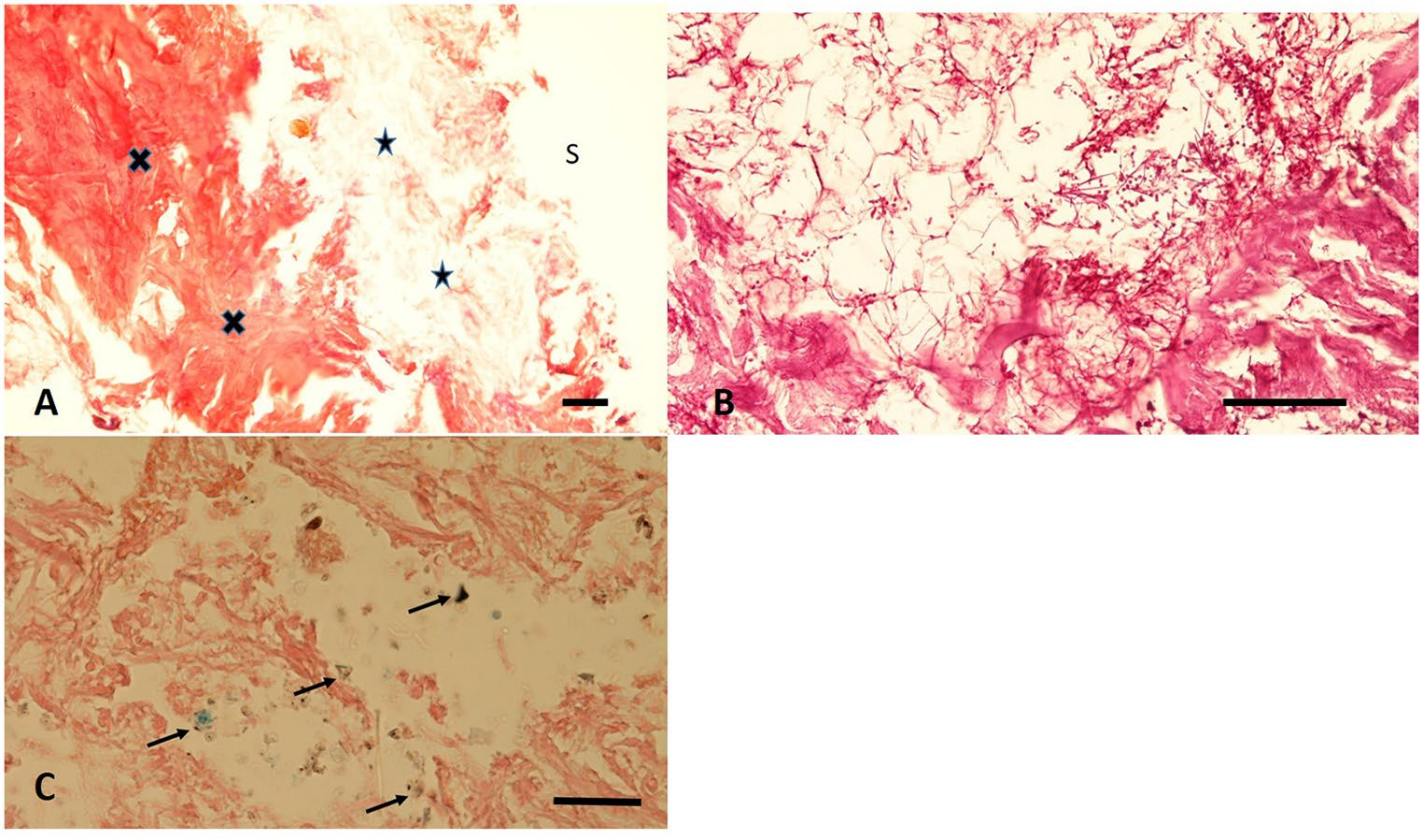
Histological features of the defect zone of the right leg: (**A**) In the overview a complete loss of the superficial epidermis is seen (S = surface), the loose reticular dermis (arrows) is distinguished from the well preserved deep dense connective tissue (crosses). (**B**) The loose upper dermis contains numerous fungal hyphae and coccoid bacteria. (**C**) In the Prussian blue stain small positive deposits are present which indicate the residues of siderophages (arrows) (**A**: H&E, **B**: PAS; **C**: Prussian blue; bar = 100 μm)

The mentioned sample was successfully rehydrated and embedded into paraffin wax for histological analysis [11]. This showed a complete absence of the epidermis whereas the connective tissue of dermis and deeper fasciae are sufficiently to well preserved (Fig. 4A). All cell nuclei were lost. The surface layer was further infiltrated with small coccoid bacteria, fungi (Fig. 4B) and occasional remnants of Diptera larvae. Beyond this, the Prussian blue stain occasionally shows small groups of strongly bluish stained positive granules suggesting remnants of siderophages within the defect margins (Fig. 4C). The presence of these grouped haemosiderin droplets is a clear evidence for minimal bleeding residues of at least a few days age proving the vitality of the defect (see discussion).

No sampling of the fabric in the areas where the overlapping discolorations are present was allowed for conservation purposes. It is therefore impossible to determine whether the textile gauze had been treated with any substances or if it was simply applied to the lesion to stop fluid drainage during the funeral procedure.

## Discussion and conclusions

The interdisciplinary investigation of a naturally mummified late 18th century infant body from the church burial ground of El Piquete (Aragon, Spain) provides evidence for an intravital skin lesion on the right leg with signs of vital reaction. The presence of siderophages which occur exclusively by an intravital enzymatic conversion of (bi-valent) haematoidin to (three-valent) haemosiderin indicates not only the vitality of the wound, but also suggests a wound age of at least several days, most probably of one to two weeks age [13, 14].

The presence of an “open” wound at the time of death of the infant is further substantiated by the selective staining of the fabric tissue overlying the ulceration zone which reveals a brown staining and most likely represents the residues of suppuration at the ulceration zone.

In general, the excellent preservation of the infant mummy is well in line with the observations on another mummy from the same burial ground [15]. There, we had shown in another well preserved infant mummy with most presumably a neurenteric duplication cyst resulting in a posterior mediastinal mass.

In the case described here, we propose that, in the pre-antibiotic era, the skin lesion became infected and, ultimately, led to the development of systemic inflammation reaction (SIRS) and finally lethal reaction such as in septicaemia. Accordingly, no further alternative internal organ pathology could be detected, a statement which is, however, strongly restricted due to post-mortem decay. To this respect it may, however, be noted that recently a study on artificially preserved infant mummies from ancient Egypt identified three (out of 21) cases with purulent infections of soft tissues/ the subcutis which also have led to systemic septicaemia [16].

Finally, we may comment on the infant mortality in late 18th century Spain. One of the main demographic characteristics of Spain during the Eighteenth till beginning Twentieth century were the high levels of infant mortality. This was a major constraint on population growth, and growth rates in Spain accordingly were even lower than in most other countries of Europe [17]. The study of childhood mortality in Spain has traditionally encountered some major obstacles. One of these is the lack of precise records. The publication of vital statistics began late and was not maintained throughout the nineteenth century. It is therefore not possible to calculate precise infant mortality rates until after 1858 [17]. However, serious estimations calculate that about 25% of children died within the first year of life with most dying of health problems that arose either during the pregnancy and/or at childbirth [18, 19]. Likewise, in Aragon – the region where our mummy comes from – a first year´s infant mortality rate of 251 deaths/ 1000 children was estimated at the end of the 18th century [20]. Even higher values were found in Spanish provinces from the inner most parts of the Hispanic peninsula [19]. A further 25% of infants succumbed in the period between 1 year and 4 years, mostly from infectious diseases [19]. These rates significantly exceeded those of other European countries, especially of Middle and Northern European countries. Infections were reported as diphtheria, typhoid fever, typhus, malaria, yellow fever (“fiebre amarilla”), pertussis (“Tos ferina”) and measles (“sarampion”) [19]. Malnutrition also had an impact on children’s mortality. In most cases, the mere term “fever” does not allow further precision of the cause of death. Within this group, SIRS or SIRS-related deaths could easily have been included, although SIRS-related deaths unfortunately were not separately recorded as the pathophysiology of the underlying bacterial infection remained unknown at that time. In this regard, our findings of a male infant mummy of 12–16 month´s age with a protracted inflammatory focus at the leg wound might be one of the few examples where we can posthumously reconstruct the cause of death of “fever”.

In conclusion, we provide here a further example of a clear paleopathological lesion most probably leading to the premature death of a historic infantile individual due to infection and systemic septicemia.

## Key points

1. Late 17th century natural infant mummy.
2. *Intra-vitam* traumatic lesion of the right lower leg.
3. Systemic infection related syndrome (SIRS) as probable cause of death.

## References

[1] Buckberry J. Missing presumed buried? Bone diagenesis and the under-representation of Anglo-Saxon children. Assemblage. 2000;5. http://ads.ahds.ac.uk/catalogue/adsdata/assemblage/html/5/buckberr.html

[2] Lewis ME. The bioarchaeology of children. Perspectives from biological and forensic anthropology. Cambridge: Cambridge Univ. Press; 2007.

[3] Nerlich AG, Bianucci R. Mummies in crypts and catacombs. In: Shin DH, Bianucci R, editors. The handbook of Mummy studies. Singapore: Springer-Nature; 2021. pp. 741–77.

[4] Aufderheide AC, Rodriguez-Martin C. The Cambridge encyclopedia of human paleopathology. Cambridge: Cambridge Univ. Press; 1998.

[5] Cockburn A, Cockburn E, Reyman T. Mummies, disease and ancient and ancient cultures. 2nd ed. Cambridge: Cambridge Univ. Press; 1998.

[6] Aufderheide AC. The scientific study of mummies. Cambridge: Cambridge Univ. Press; 2003.

[7] Ortner D, DJ. Identification of pathological conditions in human skeletal remains. 2nd ed. London: Academic; 2003.

[8] Nerlich AG, Panzer S, Wimmer J, Hamann C, Peschel OK. Adipositas and metabolic bone disorder in a 16th century Upper Austrian infant crypt mummy – an interdisciplinary palaeopathological insight into historical aristocratic life. Front Med 9:979670. 10.3389/fmed.2022.979670 PMC9643145 36388889

[9] Jardiel Badia A. El Piquete de Quinto- Testigo de la Historia. Cartoné; 2021.

[10] Panzer S, Peschel O, Haas-Gebhard B, Bachmeier BE, Pusch CM, Nerlich AG. Reconstructing the life of an unknown (ca. 500 years-old South American Inca) mummy – multidisciplinary study of a Peruvian Inca mummy suggests severe Chagas disease and ritual homicide. PLoS ONE. 2014;9:e89528. 24586848 10.1371/journal.pone.0089528 PMC3935882

[11] Grove C, Peschel O, Nerlich AG. A systematic Approach to the application of soft tissue histopathology in paleopathology. Biomed Res Int. 2015:631465. 10.1155/2015/631465 PMC4543791 26346981

[12] Schaefer M, Black S, Scheuer L. Juvenile Osteology. A laboratory and field manual. Elsevier; 2009.

[13] Betz P. Histological and enzyme histochemical parameters for the age estimation of human skin wounds. Int J Legal Med. 1994;107:60–8. 7529545 10.1007/BF01225491

[14] Betz P, Eisenmenger W. Morphometrical analysis of hemosiderin deposits in relation to wound age. Int J Legal Med. 1996;108:262–4. 8721428 10.1007/BF01369823

[15] Loynes RD, Charlier P, Perciaccante A, Gonzalez M, Begerock A, Bianucci R. Erosive effects of a posterior mediastinal mass in a 18th to early 19th c. Spanish child mummy. Sci Med Pathol. 2018;14:574–8. 10.1007/s12024-018-0013-8 30145698

[16] Panzer S, Treitl M, Zesch S, Rosendahl W, Helmbold-Doy J, Thompson RC, Zink AR. Radiological evidence of purulent infections in ancient Egyptian child mummies. Int J Paleopathol. 2002;36:30–5. 10.1016/j.ijpp.2021.12.002 34974252

[17] Ramiro Farinas D, Sanz Gimeno A. Childhood mortality in Central Spain, 1790–1960: changes in the course of demographic modernization. Continuity Change. 2000;15:235–67. 19306525 10.1017/s0268416099003537

[18] Dopico F, Rowland R. Demografía del censo de Floridablanca. Una aproximación. Rev De Hist Econ. 1990;3:591–618.

[19] Dopica F. El Impacto demográfico De las creencias. Una evaluación desde El Siglo XVIII español. Revista De Demografía Histórica. 2014;32:51–76.

[20] Ardit M. Microanálisis demográfico en larga duración: El caso de España. In: Gonzalez Portilla M, Zarraga Sangroniz K, editors. IV Congreso De La Asociación De Demografía Histórica. Pensamiento demográfico, coyuntura y microanálisis. Volume 2. Bilbao, Servicio Editorial de la UPV/EHU; 1999. pp. 253–307.

